# LRIG1 is a conserved EGFR regulator involved in melanoma development, survival and treatment resistance

**DOI:** 10.1038/s41388-021-01808-3

**Published:** 2021-05-04

**Authors:** Ola Billing, Ylva Holmgren, Daniel Nosek, Håkan Hedman, Oskar Hemmingsson

**Affiliations:** 1 Department of Surgical and Perioperative Sciences/Surgery, Umeå University, Umeå, Sweden; 2 Department of Radiation Sciences/Oncology, Umeå University, Umeå, Sweden; 3 Department of Medical Biosciences/Pathology, Umeå University, Umeå, Sweden; 4 Wallenberg Centre for Molecular Medicine, Umeå University, Umeå, Sweden

**Keywords:** Melanoma, Growth factor signalling, Differentiation, Gene expression, Gene regulation

## Abstract

Leucine-rich repeats and immunoglobulin-like domains 1 (LRIG1) is a pan-negative regulator of receptor tyrosine kinase (RTK) signaling and a tumor suppressor in several cancers, but its involvement in melanoma is largely unexplored. Here, we aim to determine the role of LRIG1 in melanoma tumorigenesis, RTK signaling, and BRAF inhibitor resistance. We find that LRIG1 is downregulated during early tumorigenesis and that LRIG1 affects activation of the epidermal growth factor receptor (EGFR) in melanoma cells. *LRIG1*-dependent regulation of EGFR signaling is evolutionary conserved to the roundworm *C. elegans*, where negative regulation of the EGFR-Ras-Raf pathway by *sma-10*/*LRIG* completely depends on presence of the receptor *let-23*/*EGFR*. In a cohort of metastatic melanoma patients, we observe an association between LRIG1 and survival in the triple wild-type subtype and in tumors with high EGFR expression. During in vitro development of BRAF inhibitor resistance, LRIG1 expression decreases; and mimics *LRIG1* knockout cells for increased EGFR expression. Treating resistant cells with recombinant LRIG1 suppresses AKT activation and proliferation. Together, our results show that *sma-10/LRIG* is a conserved regulator of RTK signaling, add to our understanding of LRIG1 in melanoma and identifies recombinant LRIG1 as a potential therapeutic against BRAF inhibitor-resistant melanoma.

## Introduction

Human cancers are frequently driven by activating mutations in *RAS* [1] or *RAF* [2] genes or in upstream receptor tyrosine kinases (RTKs) [3]. These oncogenes are activated early during tumor development and are attractive targets for treatment [1]. A majority of melanoma tumors harbor the activating *BRAF*^*V600E*^ mutation and can be treated with BRAF- and MEK-inhibitors at the metastatic stage, resulting in significantly improved survival [4, 5]. Unfortunately, early development of drug resistance hampers treatment success and is partly due to reactivation of the signaling pathway. Tumors may circumvent BRAF inhibition by overexpressing upstream RTKs [6–8], by overexpressing BRAF [9], by increased release of RTK ligands [10–12], and by increased PI3K-AKT signaling [10].

LRIG1 is a transmembrane protein [13] that functions as a tumor suppressor and a prognostic marker in many tumor types [14], including melanoma [15]. Several observations suggest that LRIG1 tumor suppression is dependent on RTK-RAS-RAF signaling: First, in mice with a *Kras*-activated background, the most frequently mutated gene involved in progression from colon adenoma to adenocarcinoma was *Lrig1* [16], suggesting that *Lrig1* suppresses early malignant transformation in this context. Second, LRIG1 downregulates several RTKs upstream of MAPK and PI3K/AKT, such as hepatocyte growth factor-receptor and epidermal growth factor receptor (EGFR) [17–20]. LRIG1 can interact with the ectodomain of EGFR and trigger its ubiquitylation and degradation in lysosomes [17, 18]. Negative regulation of EGFR by LRIG1 has been reported in glioblastoma multiforme [21] and in melanoma upon hypoxia [22]. The ectodomain of LRIG1 can be shed from cell surfaces [23] and recombinant ectodomain can suppress cancer cell proliferation both in vitro and in vivo [24, 25]. The mechanism remains elusive, but has been suggested to be both dependent [24] and independent [25, 26] of EGFR activity.

While the mammalian *LRIG* gene family has three members [27], *LRIG1-3*, *C. elegans* has a single *LRIG* homolog, *sma-10*. SMA-10 promotes bone morphogenetic protein (BMP) signaling and interacts with BMP receptors [28]. However, no phenotypes indicative of defective RTK-RAS-RAF signaling have been observed in mutants for *sma-10*.

Here, we find that *LRIG1* is downregulated during early melanoma development. Using the vulva development model in *C. elegans*, we uncover a conserved role for *sma-10/LRIG* in regulating EGFR/LET-23-RAS-RAF signaling. The direction of the effect is dependent on EGFR/LET-23 availability, suggesting that *sma-10/LRIG* acts in fine tuning of EGFR/LET-23 signaling. In melanoma patients, the survival benefit from LRIG1 depends on EGFR levels and is lost in *BRAF* and *RAS* mutant subtypes. LRIG1 is downregulated during in vitro development of BRAF inhibitor resistance, and cells that loose LRIG1, either by gene knockout or during resistance development, become sensitized to recombinant LRIG1.

## Results

### LRIG1 is negatively regulated during early melanoma development

**Fig. 1:**
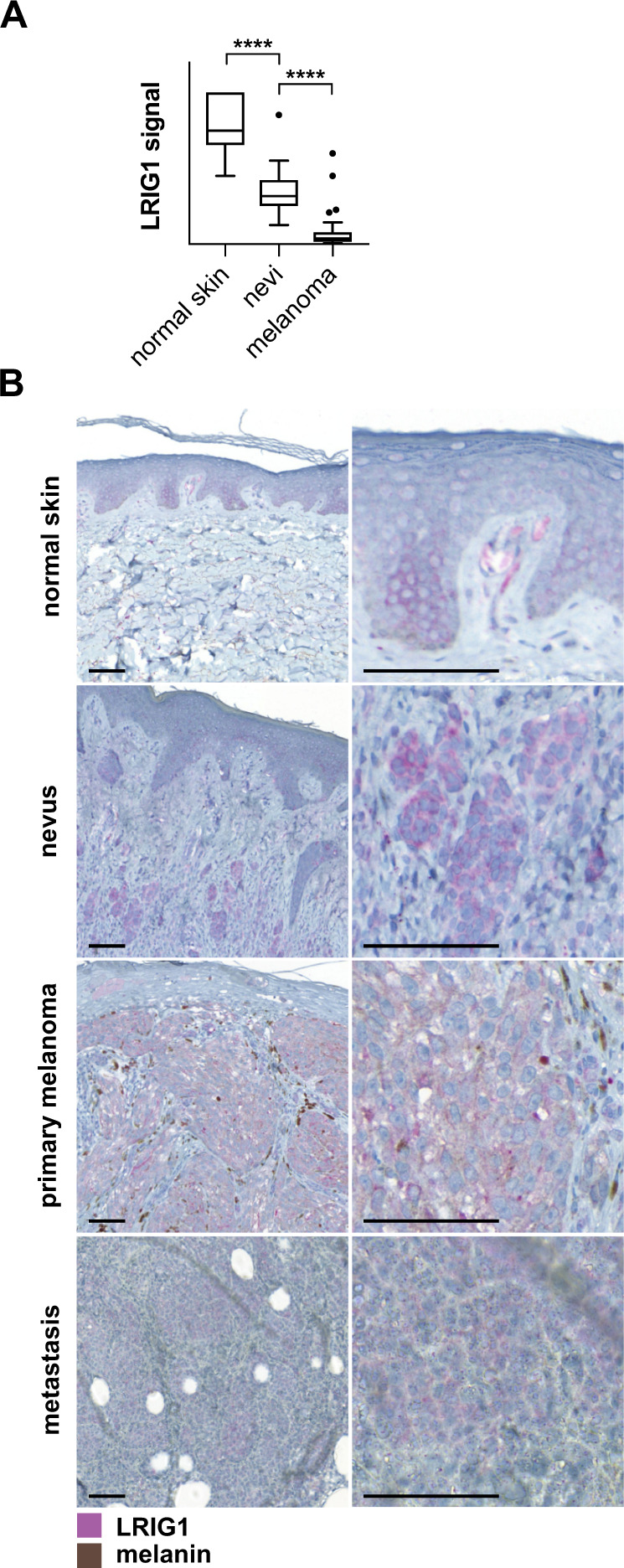
LRIG1 expression decreases during melanoma development. **A** Normalized microarray data on LRIG1 expression from normal skin tissue (*N* = 7), benign nevi (*N* = 18), and primary melanoma (*N* = 45). The boxplot shows Tukey whiskers. Statistical significance was determined with Dunnett’s multiple comparisons test. ****P < 0.0001. **B** Micrographs showing tissue sections from normal skin, nevus, primary melanoma, and metastatic melanoma stained with the Atlas anti-LRIG1 antibody. Scale bars are 100 μm.

To test the clinical relevance of LRIG1 in melanoma we first retrieved a dataset from the Gene Expression Omnibus containing microarray expression data from normal skin, benign nevi, and melanoma [29]. LRIG1 transcript levels were significantly reduced, both in melanocytic nevi compared to normal skin and during malignant transformation from nevi to melanoma (Fig. 1A). Next, we immunohistochemically stained LRIG1 in normal skin, nevi, primary melanoma, and metastases. In normal skin, we observed slightly different staining patterns with two different LRIG1 antibodies. While one antibody (Atlas) stained keratinocyte cytoplasms in the suprabasal portion of the spinous layer (Fig. 1B), the other (1A8) stained keratinocyte nuclei and throughout the spinous layer (Supplementary Fig. 1A). This prompted us to thoroughly validate both antibodies. By comparing LRIG1 knockdown and knockout cell lines to cells overexpressing an LRIG1::Flag construct, we found that both antibodies specifically detected LRIG1 (Supplementary Fig. 1C). Also, while nuclear LRIG1 has been reported in several cancer forms [30, 31] and in keratinocytes previously [32], the nuclear localization has not been validated. Here, we detected specific immunoreactivity for LRIG1 in the cell membrane, cytoplasm and nucleus, both with antibodies directed towards either side of its transmembrane domain and with an antibody against the Flag epitope (Supplementary Fig. 1B–D). Thus, LRIG1 can indeed translocate into nuclei and likely does so as a full-length protein.

Nuclear LRIG1 was absent in nevus-, primary melanoma-, and metastasis tissues, where LRIG1 instead appeared strictly cytoplasmic with both antibodies (Fig. 1B, Supplementary Fig. 1A). In nevi, the dermal epithelium was expanded, with a thickened layer of keratinocytes that had a weak LRIG1 expression. In melanoma tissue, the keratinocyte component of the epithelium was reduced and negative for LRIG1. Both LRIG1 antibodies consistently stained nevus-, primary melanoma-, and metastatic melanoma cells (Fig. 1B, Supplementary Fig. 1A).

### LRIG1 suppresses EGFR in melanoma cells

**Fig. 2:**
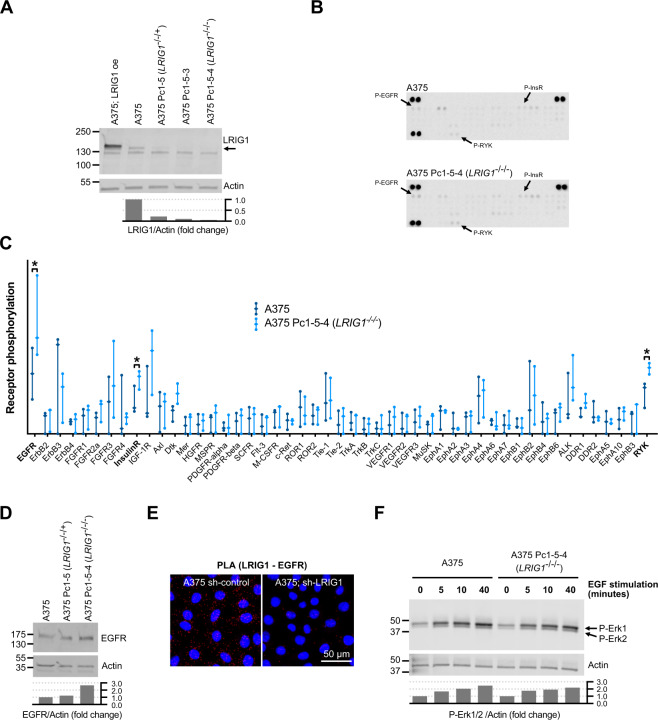
LRIG1 suppresses EGFR in melanoma cells. **A** Western blot showing LRIG1 expression in A375 parental cells and clones that were either overexpressing (oe) LRIG1 or sequentially silenced for LRIG1 using CRISPR/Cas9. Pc1-5 was sequenced and found to maintain one functional copy of LRIG1 (−/−/ + ). Pc1-5-4 was sequenced found to possess an insertion close to the splice acceptor site of LRIG1 exon 11 (−/−/−). Quantification of bands are indicated below the blot as the LRIG1/Actin fold change from A375. **B** A representative phospho-RTK array analysis on lysates from A375 cells and LRIG1 knockout A375 Pc1-5-4 cells. Duplicate spots for phospho-EGFR, phospho-RYK, and phospho-insulin receptor are indicated. **C** Quantified phospho-RTK array data. The graph shows all datapoints and distribution range from three independent experiments. Statistical significance between A375 and A375 Pc1-5-4 lysates was determined using paired, two-tailed Student’s *t* tests for each phospho-RTK. *P < 0.05. **D** Western blot showing EGFR expression in A375 cells and in LRIG1 knockdown and knockout subclones. Quantifications are indicated as the EGFR/Actin fold change from A375. **E** Micrographs showing EGFR-LRIG1 proximity ligation assay micrographs in A375 cells treated with shRNA control (sh-control) or shRNA against LRIG1 (sh-LRIG1). **F** Western blot analysis of phospho-ERK1/2 protein in A375 cells and LRIG1 knockout A375 Pc1-5-4 cells after stimulation with 10 ng/ml EGF for the indicated time. Quantifications are indicated as the p-Erk1/2/Actin fold change from untreated A375 cells.

LRIG1 downregulates RTKs in several cancers. To investigate if the same was true in melanoma cells, we generated LRIG1 knockout cells from the melanoma cell line A375 (Fig. 2A) and screened for altered levels of phosphorylated RTKs using antibody arrays (Fig. 2B, C and Supplementary Fig. 2). In LRIG1 knockout cells, we detected significantly increased signals for phospho-insulin receptor, phospho-RYK, and phospho-EGFR, and we decided to further investigate the LRIG1-EGFR interplay. The increased phospho-EGFR expression likely depended on increased protein levels rather that increased relative phosphorylation, since sequential LRIG1 knockdown resulted in sequentially increased levels of EGFR (Fig. 2D). While we were unable to confidently demonstrate a physical interaction between endogenous LRIG1 and EGFR by co-immunoprecipitation (not shown), we noted that LRIG1 and EGFR appeared to localize closely by virtue of a proximity ligation assay (Fig. 2E). Curiously, the increased phospho-RTK levels observed in LRIG1 knockout cells did not appear to affect downstream ERK1/2 signaling or sensitivity to EGF stimuli (Fig. 2F).

### LRIG1 is a conserved regulator of RTK-RAS-RAF signaling

**Fig. 3:**
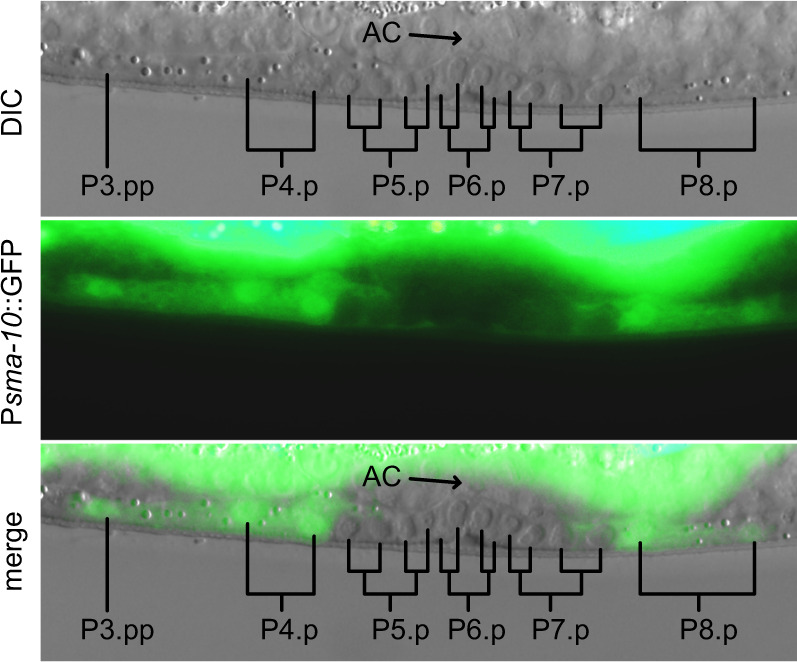
LRIG/SMA-10 is only expressed in non-induced vulva precursor cells during *C. elegans* vulva development. Micrograph showing developing vulva tissue in a stage L3 hermaphrodite. The first two rounds of mitosis are indicated. GFP expression from the translational fusion P*sma-10*::GFP is present in P3.p, P4.p, and P8.p progeny and absent in P5.p, P6.p, and P7.p progeny. *AC* Anchor Cell.

LRIG1 regulated multiple RTKs in A375 cells simultaneously, making detailed pathway analysis difficult. By contrast, in the *C. elegans* vulva development model, the well-conserved EGFR-homolog LET-23 is the sole RTK responsible for pathway activation. Here, the inductive signal from EGFR/LET-23 governs cell specification of six vulva precursor cells (p-cells) in developing larvae. In wild-type animals, the central p-cells p5.p, p6.p, and p7.p are induced to form vulva tissue while EGFR/LET-23 signaling is suppressed in the lateral p3.p, p4.p, and p8.p cells, which will avoid vulval fates. To determine if the *C. elegans* LRIG-homolog *sma-10* could be involved in vulval development, we first examined the expression pattern from a translational GFP reporter under control of the *sma-10* promoter (Fig. 3). Strikingly, LRIG/SMA-10 was expressed only in the lateral p-cells cells that should not be induced, whereas LRIG/SMA-10 was absent from the central p-cells that should be induced by EGFR/LET-23 (Fig. 3).

**Fig. 4:**
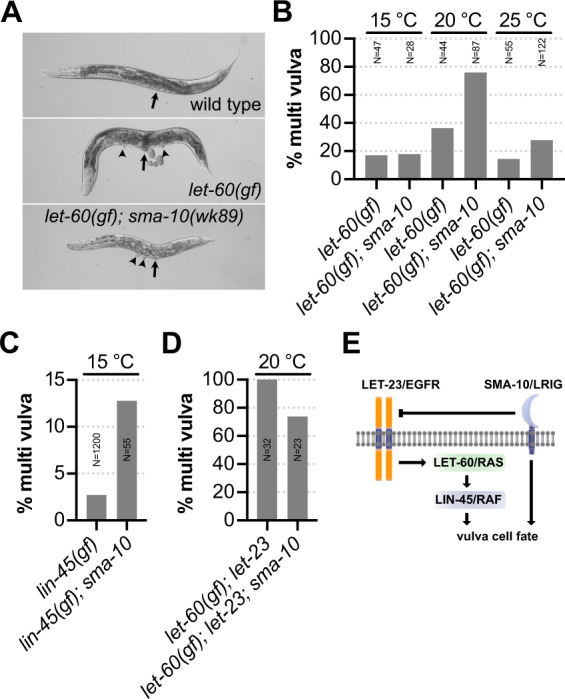
LRIG/SMA-10 is a regulator of EGFR/LET-23 signaling in *C. elegans*. **A** Micrographs showing body morphology of indicated genetic backgrounds. Arrows point to fully developed vulva structures and arrowheads indicate the presence of additional pseudovulvae, i.e., the multi vulva phenotype (Muv). **B** Animals of *let-60(n1046gf)* and *let-60(n1046gf); sma-10(wk89)* genotypes respectively were grown to the adult stage at the indicated temperature and scored for presence of the Muv phenotype under high power microscopy. **C** *Lin-45* gain of function mutants [*lin-45(gf)*] were grown alongside *lin-45(gf); sma-10(wk89)* double mutants and scored for the Muv phenotype. Both genotypes threw 0% Muv progeny at 20 °C (*N* = 61–68) and 25 °C (*N* = 58–86). **D** Percentage of animals scored with the Muv phenotype at 20 °C in *let-60(n1046gf); let-23(mn23)* double mutants and *let-60(n1046gf); let-23(mn23); sma-10(wk89)* triple mutants, respectively. **E** Model depicting the regulatory circuit of SMA-10/LRIG during *C. elegans* vulval induction. LET-23/EGFR signals through the canonical RTK-RAS-MAPK pathway to induce vulva development. The primary function of *sma-10*/LRIG in vulva development is inhibition of LET-23/EGFR. The inhibitory effect from SMA-10/LRIG is entirely dependent on LET-23/EGFR; and in its absence, SMA-10/LRIG instead promotes vulval induction.

A role for LRIG/SMA-10 in vulva development and RTK-Ras-MAPK signaling has not been demonstrated. Indeed, *sma-10(wk89)* null mutants showed wild-type vulva development at 15, 20, and 25 °C (*N* = 100 for each temperature). However, to our knowledge, this has not previously been investigated in a sensitized genetic background. The *G13E* mutation in the worm *RAS* homolog mutant *let-60(n1046gf)* favors an activated configuration of the RAS/LET-60 GTPase [33]. A fraction of such mutants will develop a multi vulva (Muv) phenotype, since lateral p-cells that normally avoid vulval fates are erroneously induced and develop into pseudovulvae (Fig. 4A). In the *RAS/let-60(n1046gf)* background, animals harboring the null mutation *sma-10(wk89)* displayed an increased ratio of Muv animals (Fig. 4A, B). This shows that *sma-10/LRIG* suppresses the inductive signal during vulva development. The effect was temperature-sensitive and evident only at 20 °C and 25 °C.

Using CRISPR-Cas9-directed homologous recombination, we then generated a substitution mutation in the RAF*/lin-45* gene, resulting in a V627E substitution, analogous to the V600E mutation in human *BRAF*. This first RAF/*lin-45* gain of function mutant in *C. elegans* displayed a low penetrance Muv phenotype that was only present at 15 °C and only in 2.4% (*N* = 1200) of the animals (Fig. 4C). The *sma-10(wk89)* mutation enhanced the Muv phenotype of *RAF*/*lin-45* gain of function mutants (Fig. 4C).

*EGFR/let-23* is required for survival at an early larval stage. To test if *sma-10* affected this, we compared hypomorph *let-23(n1045hyp)* mutants with *let-23(n1045hyp); sma-10(wk89)* double mutants but found *sma-10* to be dispensable for early larval lethality (Supplementary Fig. 3). The lethality of *EGFR/let-23(mn23)* null mutants can be rescued by excessive downstream signaling from the *RAS/let-60(n1046gf)* allele [34]. However, the only surviving animals are those with a strong signal from the *RAS/let-60(n1046gf)* allele. As a result, the baseline for the Muv phenotype was 100% in *RAS/let-60(n1046gf); EGFR/let-23(mn23)* double mutants (Fig. 4D). Strikingly, the Muv phenotype in this background was suppressed by the *sma-10(wk89)* mutation (Fig. 4D). This demonstrates two things: First, suppression of Ras-MAPK signaling by SMA-10/LRIG depends on LET-23/EGFR availability. Second, in the absence of LET-23/EGFR, SMA-10/LRIG instead promotes vulva cell fates, either downstream of LET-23/EGFR or in a yet to be determined, parallel pathway (Fig. 4E).

### Association between survival and LRIG1 expression appears EGFR-dependent

To investigate if LRIG1 could be correlated to melanoma survival, we extracted a dataset from The Cancer Genome Atlas (TCGA) containing clinical data and RNAseq data from primary and metastasized melanoma tumors. This yielded a cohort of 389 patients with sequencing data from primary tumor tissue (*N* = 90) and metastases (*N* = 299). Using observed survival interval (OBS) [35] in univariable COX regression analyses we found that *LRIG1* expression appeared dispensable for survival when measured in both primary tumors (hazard ratio = 0.807, 95% confidence interval = 0.348–1.870, *p* = 0.617) and metastases (hazard ratio = 0.828, 95% confidence interval = 0.605–1.132, *p* = 0.236).

**Fig. 5:**
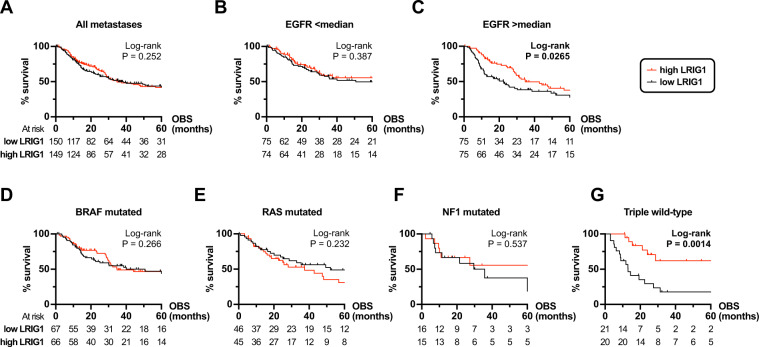
Survival association for *LRIG1* in melanoma depends on *EGFR* expression and on molecular subtype. Kaplan–Meier estimates based on RNAseq data, mutation status, and observed survival (OBS) data extracted from a TCGA data set on metastasized melanoma patients: **A** All 299 patients in the metastatic melanoma cohort with sequenced tissue from TCGA was divided into *LRIG1* high/low groups based on the median. This cohort was further divided in two sub-cohorts at the median for *EGFR* tumor tissue expression. The resulting sub-cohorts of patients with (**B**) low *EGFR* expression or (**C**) high *EGFR* expression were then analyzed based on *LRIG1* expression and OBS using Kaplan–Meier estimates. **D**–**G** Similarly, using the same dataset, we extracted patient samples with available mutational data (*N* = 296). We then divided those into molecular subtypes as defined previously [36]: **D** Patients with activating V600 or K601 mutations in *BRAF*. **E** Patients with activating G12, G13, or Q61 mutations in *HRAS*, *KRAS*, or *NRAS*. **F** Patients with inactivating mutations in *NF1*. **G** Triple wild-type patients without any of the above mutations.

However, since our genetic analyses in *C. elegans* indicated that negative regulation of RAS-MAPK signaling by LRIG/SMA-10 required the presence of EGFR/LET-23, we hypothesized that a tumor-suppressive function of LRIG1 may be exerted at the EGFR level. To test this, we first used Kaplan–Meier estimates to analyze the OBS in the metastasized patient cohort (*N* = 299). As expected from the previous analysis, there was no difference in survival between the *LRIG* high vs. low groups (Fig. 5A). We then analyzed if *EGFR* availability corresponded with OBS in the *LRIG1* groups. By dividing the metastatic cohort based on *EGFR* expression, we found that high *LRIG1* expression associated with improved survival only in the cohort with high *EGFR* expression (Fig. 5B, C).

Previous work have classified four melanoma subtypes based on mutations in genes affecting RAS-RAF-MAPK signaling [36]. These are (1) mutant *BRAF* (V600, K601), (2) mutant *H/N/K RAS* (G12, G13, Q61), (3) mutant *NF1*, and (4) triple wild-type. We next divided the metastatic cohort with available mutational status (*N* = 296) into each proposed subtype (Supplementary Fig. 4). Strikingly, while *LRIG1* expression did not associate with survival in *BRAF* (*N* = 133)*, RAS* (*N* = 91), or *NF1* (*N* = 31) subtypes (Fig. 5D–F), high *LRIG1* associated with better survival in triple wild-type patients (*N* = 41, Fig. 5G). In summary, high expression of *LRIG1* associated with better survival in patients with high *EGFR* expression and in the triple wild-type subtype, which is devoid of pathway-activating mutations downstream of the RTK.

### Recombinant LRIG1 suppresses proliferation in LRIG1 knockout cells

**Fig. 6:**
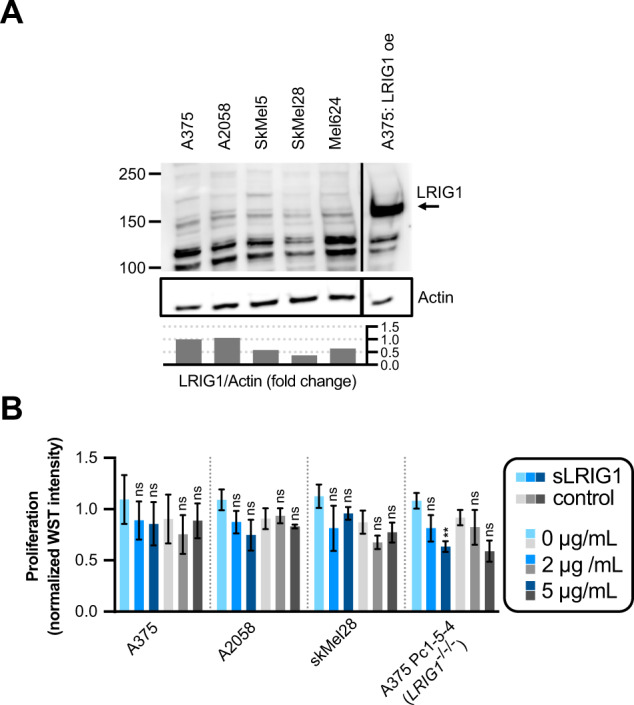
LRIG1 knockout cells are sensitive to treatment with recombinant LRIG1 ectodomain. **A** Western blot analysis of LRIG1 protein expression in a panel of melanoma cell lines. The blot has been cropped vertically for clarity. Quantifications are indicated as the LRIG1/Actin fold change from A375. **B** Proliferation (WST intensity) after treatment with recombinant protein in four different cell lines. Normalization between experiments was done separately for each individual cell line by division with the mean WST intensity value of the two untreated (0 μg/mL) conditions. Concentrations of supplemented proteins are indicated for recombinant LRIG1 ectodomain (sLRIG1, blue bars) and His-MBP-Strep control protein (gray bars). Statistical significance vs. 0 µg/mL protein was tested using a two-way ANOVA with a Tukey’s multiple comparisons test.

The ectodomain of LRIG1 can be shed from cell surfaces and affect cells in a paracrine fashion [23]. Also, recombinant LRIG1 ectodomain suppresses cell proliferation in glioma cells, both in vitro and in vivo [25]. To test if recombinant LRIG1 ectodomains (sLRIG1) could suppress melanoma cell proliferation, we exposed a panel of melanoma cell lines with variable LRIG1 expression (Fig. 6A) to sLRIG1 but detected no significant effects on proliferation (Fig. 6B). *LRIG1* knockout A375 cells did, however, become sensitized to sLRIG1, which suppressed cell proliferation at 5 µg/mL (Fig. 6B).

### Vemurafenib-resistant cells loose LRIG1, gain EGFR and are sensitized to recombinant LRIG1

**Fig. 7:**
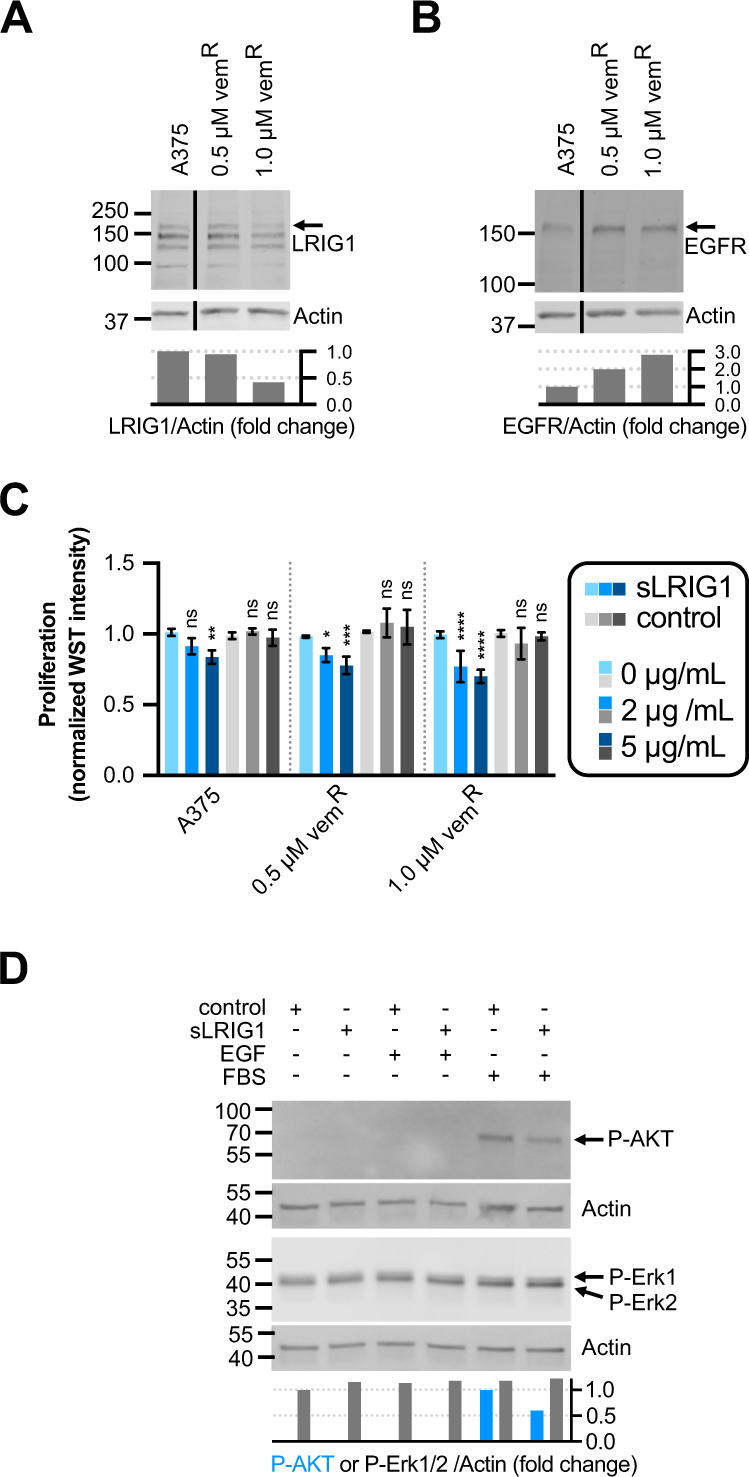
BRAF inhibitor-resistant cells lose LRIG1, gain EGFR and become sensitized to recombinant LRIG1 ectodomain treatment. **A**, **B** Western blot showing protein expression in naïve A375 cells and two resistant lines with increasing levels of vemurafenib resistance probed for (**A**) LRIG1 and (**B**) EGFR. The blots have been cropped vertically for clarity. Quantifications are indicated as the LRIG1/Actin or EGFR/Actin fold change from A375 respectively. **C** Proliferation (normalized WST intensity) after treatment with recombinant protein in four different cell lines. Normalization and statistics were performed as in Fig. 6B. Concentrations of supplemented proteins are indicated for recombinant LRIG1 ectodomain (sLRIG1, blue bars) and His-MBP-Strep control protein (gray bars). **D** Western blots on cell lysates form vemurafenib-resistant cells, stimulated as indicated and probed for phospho-AKT and phospho-Erk1/2. Quantifications are indicated as the phospho-AKT/Actin fold change from FBS-stimulated cells treated with control peptide or as the phospho-Erk1/2/Actin fold change from serum-starved cells treated with control peptide.

Pharmacological BRAF inhibition is an established treatment for *BRAF*^*V600E*^-positive melanoma. A common resistance mechanism involves upregulation of EGFR signaling [37]. By exposing A375 cells to increasing levels of the BRAF inhibitor vemurafenib, we generated resistant lines (Supplementary Fig. 5C). Similar to the sequential knockout of LRIG1 in A375 cells (Fig. 2E), resistant cells lost expression of LRIG1 protein (Fig. 7A) and gained expression of EGFR protein (Fig. 7B). Next, we transduced a full-length LRIG1 construct under the control of a doxycycline-inducible promotor into BRAF inhibitor-resistant cells (Supplementary Fig. 5D). However, induction of ectopic LRIG1 expression had no effect on BRAF inhibitor resistance (Supplementary Fig. 5E). Also, LRIG1 knockout did not affect proliferation, vemurafenib tolerance, or the ability to acquire resistance to vemurafenib (Supplementary Fig. 5A–C).

Since *LRIG1* knockouts became sensitized to treatment with sLRIG1, and vemurafenib-resistant cells lost their LRIG1 expression, we tested resistant cell lines for sLRIG1 sensitivity. Strikingly, treatment with sLRIG1 suppressed proliferation at both 2 and 5 µg/mL (Fig. 7C). The treatment effect appeared to increase with increased level of vemurafenib resistance. While treatment with 5 µg/mL sLRIG1 in resistant cells had no effect on ERK activation (Fig. 7D), it suppressed phospho-AKT in FBS-stimulated cells (Fig. 7E). In conclusion, vemurafenib resistance in A375 cells associated with loss of LRIG1, gain of EGFR and sensitivity to recombinant LRIG1, which suppressed phosphorylation of AKT.

## Discussion

We have shown that LRIG1 is a conserved EGFR regulator that is lost in early melanoma development. In *C. elegans*, the sole LRIG homolog SMA-10 can both promote and suppress the LET-23/EGFR signaling pathway, where suppression is completely dependent on the presence of LET-23/EGFR. In metastasized melanoma patients, *LRIG1* expression in metastases predicts survival when *EGFR* levels are high and in the triple wild-type subtype. By contrast, activating downstream mutations in *BRAF, RAS*, or *NF1* negates the survival benefit conferred by high *LRIG1* expression. Even so, *BRAF*^*V600E*^ mutant cells that become resistant to the BRAF inhibitor vemurafenib loose LRIG1, gain EGFR and become increasingly sensitive to treatment with recombinant LRIG1 in vitro.

Decreased *LRIG1* expression was previously shown to associate with worse survival in metastatic melanoma [15]. Two other studies in mice showed that LRIG1 can suppress both melanoma tumor growth [38] and invasion [22]. The current study shows that *LRIG1* expression is associated with survival in an *EGFR*-dependent manner and that the association is lost in melanoma subtypes characterized activating mutations downstream of EGFR. Interestingly, the molecular subtype of melanoma patients has previously not been associated with survival [36]. Here we report that advanced staged, triple wild-type melanoma patients have a survival benefit from high *LRIG1* expression.

From an evolutionary perspective, the previous lack of evidence for an RTK regulating function in ancestral LRIG proteins has remained puzzling. *C. elegans* SMA-10, Drosophila Lambik and human LRIG1 all bind and regulate BMP receptors [28, 39], but roles in RTK signaling have so far only been described for mammalian LRIGs. In *C. elegans*, we find that the sole LRIG homolog SMA-10 indeed functions in RTK signaling. The direction of the effect was completely dependent on LET-23/EGFR, which is the sole RTK required for vulval cell specification. In presence of the RTK, SMA-10 suppressed the pathway while in the absence of the RTK, SMA-10 instead promoted the pathway. Although the promoting signal needs further characterization and may depend on lateral signaling and cell non-autonomous effects, it remains clear that pathway suppression by LRIG/SMA-10 completely depends on the RTK. In addition, the activity of LRIG/SMA-10 appeared temperature-sensitive. Neither temperature-dependence nor the positive signaling component in the absence of RTK substrate has been demonstrated for LRIG proteins previously. This context-dependence implies a role for LRIG proteins in RTK signaling fine-tuning, similar to what has been observed for other RTK-Ras-Raf pathway regulators [40, 41]. Although beyond the scope of this study, the regulatory role of LRIG proteins on RTK signaling could possibly depend on their effects on BMP signaling or on the reported function for SMA-10/LRIG in membrane receptor trafficking [39].

In this study, phospho-RTK arrays showed upregulation of phospho-EGFR, phospho-insulin receptor, and phospho-RYK in LRIG1-depleted cells. To our knowledge, LRIG1-dependent effects on the insulin receptor or on RYK has not been reported before and need further validation. Most other tested phospho-RTKs also trended towards increased levels, with the exception of ERBB3. The apparent lack of connectivity between RTK phosphorylation and downstream ERK1/2 phosphorylation in A375 cells could possibly depend on their downstream activating *BRAF*^*V600E*^ mutation. On the other hand, the analog *RAF/lin-45(gf)* mutation in *C. elegans* was sensitive to upstream signaling. The same was true for *RAS/let-60(gf)*. These observations suggest that gain of function mutant RAS and RAF proteins are preferentially activated but still able to respond to upstream signaling. This may possibly explain why upregulated RTK signaling can mediate BRAF inhibitor resistance through pathway reactivation in melanoma cells, even though the activating mutation under treatment is *BRAF*^*V600E*^. The vemurafenib-resistant cell lines generated in vitro showed upregulation of EGFR and downregulation of LRIG1. During early resistance development (0.5 µM), upregulation of EGFR appeared more prominent than loss of LRIG1 (Fig. 7A, B). However, these early resistance dynamics were not consistent between repeated experiments and the sequence of events remains to be characterized in detail. Forced expression of full-length LRIG1 had no effect on proliferation in neither BRAF inhibitor-resistant cells nor LRIG1 knockout cells but treatment with recombinant LRIG1 ectodomain suppressed proliferation. We thus speculate that the location of LRIG1 may be important for its function. Indeed, the ectodomain of LRIG1 can be shed from full-length LRIG1 after proteolytic cleavage both in vitro and in vivo [23] and recombinant LRIG1 ectodomain has been demonstrated to suppress tumor growth in a mouse glioma model [25]. Ectopic overexpression of EGFR is sufficient to cause vemurafenib resistance in melanoma cells in vitro [37]. It has also been demonstrated that EGF can promote AKT activity in resistant cells and that combination treatment with vemurafenib and PI3K-AKT signaling inhibitors can suppress the development of vemurafenib resistance in mice [10]. In our hands, EGF stimulation alone was not sufficient to detect phospho-AKT in resistant cells. However, in FBS stimulated cells, both proliferation and AKT phosphorylation were suppressed by recombinant LRIG1. We speculate that recombinant LRIG1 may suppress proliferation by inhibiting RTK-dependent activation of the PI3K-AKT signaling pathway in vemurafenib-resistant melanoma cells.

In human tissue, both nevi cells and melanoma cells showed cytoplasmic LRIG1 immunoreactivity. In normal skin keratinocytes and in LRIG1 overexpressing cell lines, LRIG1 occasionally showed a nuclear immunoreactivity. Although nuclear immunoreactivity for LRIG1 has been reported previously [30–32], we validated it here by immunocytochemistry. Nuclear LRIG1 was detected with antibodies directed both towards the endo- and the ectodomain, indicating that nuclear LRIG1 is likely the full-length protein. The nuclear localization appeared to be conditional, depending on both the physiological context as well as on technical parameters, including antibodies and fixation protocols. Interestingly, RTKs such as EGFR also occasionally localizes to nuclei, where they can bypass canonical signal transduction and promote gene transcription [42]. Similarly, the LRIG1-interacting protein LMO7 [43] has been reported to shuttle between the cytoplasm and the nucleus [44, 45]. We speculate that nuclear LRIG1 might regulate such interactors in the nucleus. However, the biological relevance and the function of nuclear LRIG1 remains to be characterized. Further, we found that LRIG1 expression appeared to decrease with keratinocyte hyperproliferation in nevi tissue, similar to what was previously observed in psoriatic skin lesions [32]. This finding may be relevant for early melanoma development since keratinocytes are known to suppress melanocyte proliferation and migration through cell–cell contacts and paracrine regulation of RTK signaling [46]. We also note that the keratinocyte hyperproliferation that associated with loss of LRIG1 resembles a phenotype described in Lrig1 knockout mice [47].

In summary, LRIG1 is a conserved regulator of EGFR signaling that is negatively regulated during early melanoma development and that associates with survival in an EGFR-dependent manner at the metastatic stage. BRAF inhibitor-resistant melanoma cells are sensitive to recombinant LRIG1, which should be further explored as a potential therapy to combat BRAF inhibitor-resistance.

## Materials and methods

### Datasets

Normalized microarray data (GDS1375) from seven normal skin tissue specimens, 18 benign skin nevi, and 45 primary melanomas [29] was retrieved through the Gene Expression Omnibus (https://www.ncbi.nlm.nih.gov/geo/).

We downloaded a TCGA dataset (*N* = 481) with log2(x + 1) transformed and RSEM-normalized RNAseq counts, mutation calls and clinical data from UCSC Xena (http://xena.ucsc.edu) [48] in August 2020. Exclusion criteria were (1) missing data regarding expression, survival or days to submitted specimen; (2) Neoadjuvant treatment; (3) Non-melanoma malignancy during follow-up; (4) Follow-up time <60 days; (5) Without new tumor during follow-up and diseased; (6) Sample type additional metastasis, solid tissue normal or missing. To compensate for the out of step issue where overall survival starts from the primary diagnosis and RNAseq data are acquired at the time of sampling, we generated a surrogate survival time, calculated as observed survival (OBS) = overall survival − days to submitted specimen [35].

When analyzing survival in advanced disease stages stratified on *BRAF*, *(H/N/K)RAS*, and *NF1* mutations, these were grouped into four melanoma subtypes [36]. Three patients lacked mutation data and were excluded from this analysis.

### Cell lines

Melanoma cell lines A375, A2058, SkMel5, SkMel28, Mel624 were gifts from S. Vagner and C. Robert (Gustave Roussy, Villejuif, France) who obtained all cells except Mel624 from ATCC (Manassas, VA, USA). The non-small cell lung cancer cell line H1975 was obtained from ATCC. Melanoma cell lines were grown in DMEM with 10% FBS and H1975 was grown in RPMI + 10% FBS. All cell lines were tested for mycoplasma (GATC/ Eurofins Genomics, Ebersberg, Germany) and authenticated through short-tandem repeats profiling at ATCC or IDEXX BioAnalytics (Kornwestheim, Germany). Generation of LRIG1 knockdown, knockout and overexpressing cells is described in detail in the Supplementary Methods section. Vemurafenib-resistant lines were generated by exposing A375 cells to increasing concentrations of PLX4032 (Selleckchem) in steps of 0.05 µM per passage. After reaching resistance to 0.5 µM, steps were increased to 0.1 µM and after 1.0 µM, steps were increased to 0.5 µM. The selection process was carried out within 23 passages.

### Immunohistochemistry

Five µm tissue sections were stained for LRIG1 protein using a staining machine (Ventana Medical Systems, Tucson, AZ, USA). Antigen retrieval was performed in citrate buffer at pH 6.0 for eight minutes at 91 °C. The Ventana Universal Alkaline Phosphatase Red Detection Kit was used, and sections were counterstained with Mayer’s hematoxylin. Normal human skin, liver and colon served as positive controls. Tissue slides were visualized using a Panoramic 250 Flash scanner (3DHISTECH Ltd, Budapest, Hungary) and analyzed by a pathologist (D.N.).

### Antibodies

The antigen peptides for all LRIG1 antibodies used are indicated in Supplementary Fig. 1B. For immunohistochemistry: Anti-LRIG1 antibody (Atlas Antibodies AB, Bromma, Sweden, catalog no. HPA011846), 4.0 mg/ml. The 1A8 anti-LRIG1 antibody is described in the Supplementary Materials. For western blots: EGFR #2232, 1:1000; Phospho-p44/42 MAPK #4370, 1:1000; Phospho-AKT #4060, 1:1000 (Cell Signaling Technology Inc, Danvers, USA); Anti-Actin #ACTN05/C4, 1:3000 (Abcam, Cambridge, UK). Anti-LRIG1 Vina [49], 1:1000 (Agrisera AB, Vännäs, Sweden). Secondary antibodies: IRDye^®^ 680RD Donkey anti-Mouse #926-68072, 1:15000; IRDye^®^ 800CW Goat anti-Rabbit #926-32211, 1:15000 (LI-COR, Lincoln, NE, USA).

### Western blots

Cells were washed twice in ice-cold PBS and lysed for 5 min in RIPA buffer (Thermo Fisher Scientific, Waltham, MA, USA), supplemented with Complete protease inhibitor cocktail and Phosstop phosphatase inhibitors (Roche Applied Science, Penzberg, Germany). Lysates were spun down at 4 °C and supernatants were collected. Protein concentrations were determined with the BCA method. Samples were diluted in Laemmli buffer + ß-mercaptoethanol and boiled at 95 °C for 5 min. Twenty µg/sample were run out on either 4–20% TGX polyacrylamide gels (BioRad, Hercules, USA) or 3–8% tris-acetate gels (Fisher Scientific, Waltham, MA, USA), and blotted onto TGX PVDF membranes (BioRad, Hercules, USA). Blots were blocked for 1 h at room temperature in Odyssey TBS blocking buffer (Li-Cor Biosciences, Lincoln, USA) and were then incubated with primary antibodies in the same buffer overnight at 4 °C. After washing in TBST, blots were incubated with secondary antibodies for 2 h at room temperature. Blots were imaged on an Azure c600 machine (Azure Biosystems, Dublin, USA) and quantified using Image Studio Light software v5.2.5 (Li-Cor Biosciences, Lincoln, USA).

### Phospho-receptor tyrosine kinase arrays

Cells were seeded at ~33% confluency and grown overnight in DMEM + 10% FBS. Cells were then reseeded at 90,000 cells/2 mL into 6-well plates and grown in DMEM + 10% FBS. Cells were grown for 48 h and then lysed and treated according to the protocol for RnD’s Human Phospho-RTK Array Kit (R&D Systems, Inc, Canada). Imaging was done on an Azure c600 and quantifications were done using Gilles Charpentier’s Protein Array Analyzer plugin in Fiji v2.0.0 software [50].

### Cell proliferation experiments

Cell cultures were grown until ~80% confluent, trypsinated and re-seeded at 3000 cells/well in 96-well plates. After an overnight incubation, cells were supplemented with test media (vemurafenib, doxycycline, control His-MBP-Strep peptide or recombinant LRIG1 ectodomain) and were incubated for an additional 48 h. Cell densities were measured using the WST cell proliferation reagent according to the manufacturer’s instructions (Roche Applied Science, Penzberg, Germany).

### Proximity ligation assay

A375 sh-control and A375 shLRIG1 cells were grown on coverslips, washed in PBS and fixated in water-free acetone at −20 °C. Fixed cells were blocked in PBS with 5% FBS and 0.05% Saponin for 30 min in 37 °C. Samples were then incubated at 4 °C overnight with the primary antibodies mouse anti-LRIG1 1A8 1:300 and rabbit anti-EGFR #4267 1:50 (Cell Signaling Technology Inc, Danvers, USA). After washing twice in PBS, cells were incubated with anti-mouse and anti-rabbit PLA probes and treated according to the manufacturer’s instructions (Sigma-Aldrich Sweden AB). PLA and DAPI-stained cells were visualized on a Zeiss 710 confocal microscope.

### *C. elegans* strains and experiments

Bristol N2 was the wild-type strain used in all *C. elegans* experiments. Worms were maintained at 20 °C on NGM agar plates seeded with *E. coli* OP50. The procedure for generating the *lin-45gf* allele is described in the supplementary methods section. Other mutant alleles used in this study were *sma-10(wk89), let-23(n1045hyp), let-23(mn23)/*mIn1, and *let-60(n1046gf)*. Vulval phenotypes were scored in adult animals raised at the indicated temperature and analyzed using an Olympus BX51 microscope. Larval lethality was determined by transferring mothers to new plates each day and scoring dead and viable larvae 72 h later. The BC14516 [sEx14516 (P*sma-10*::GFP)] strain was a kind gift from Richard Padgett (Rutgers University, NJ, USA). These animals were grown at 20 °C until the L3 stage. GFP-positive animals were visualized using an Olympus BX51 microscope.

### Statistics

Unless otherwise stated, all error bars indicate standard deviations. Microarray data were compared with a Dunnett’s multiple comparisons test and cell line and animal experiments were analyzed with Student’s *t* tests or two-way ANOVA with a Tukey’s multiple comparisons test, all using Graphpad Prism v7 software. Univariable cox regression was performed using STATA v16 software. Kaplan–Meier estimates were generated using Prism v7 and statistical significance was calculated using log-rank (Mantel-Cox) tests.

## Supplementary information

Supplementary figures

Supplementary methods and figure legends
