# Supplementary material for: LRIG1 is a conserved EGFR regulator involved in melanoma development, survival and treatment resistance
Source: Oncogene. 2021 May 4;40(21):3707–18. doi: 10.1038/s41388-021-01808-3 (PMC8154585; doi:10.1038/s41388-021-01808-3)

SUPPLEMENTARY FIGURE 1

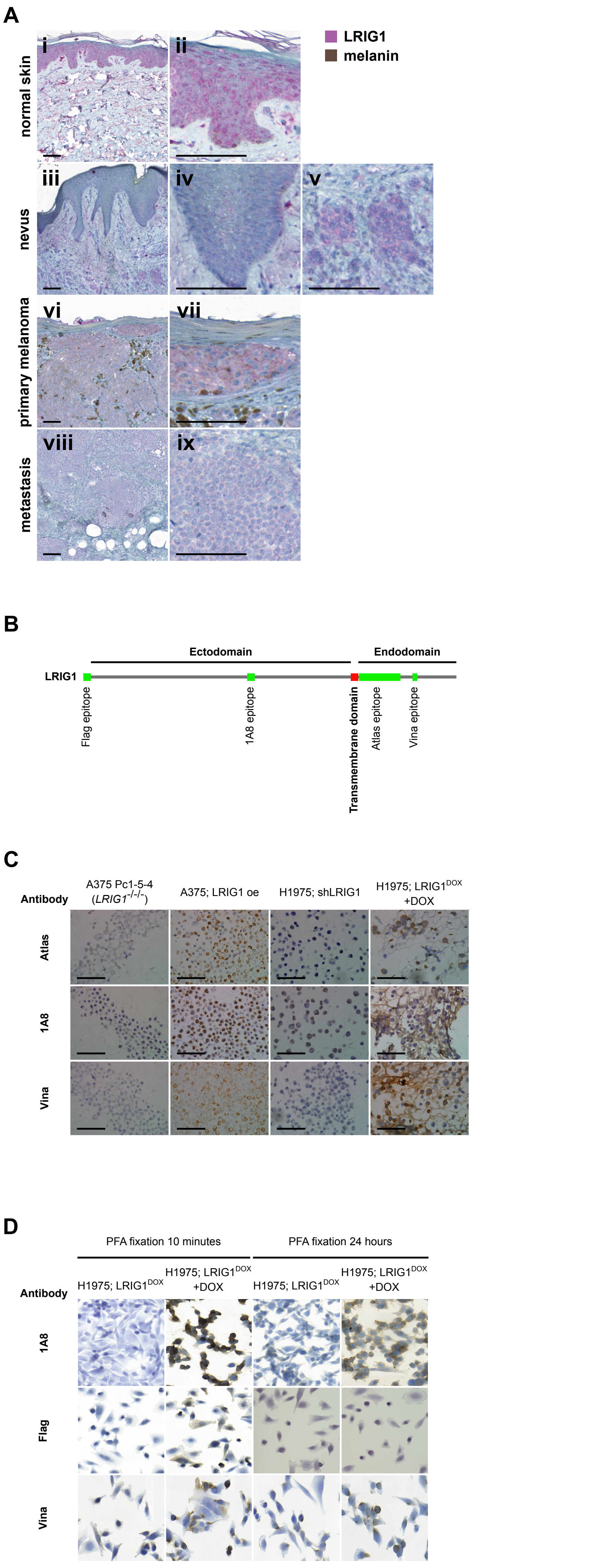

SUPPLEMENTARY FIGURE 2

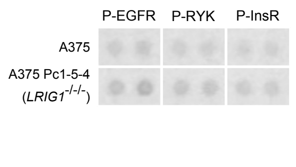

SUPPLEMENTARY FIGURE 3

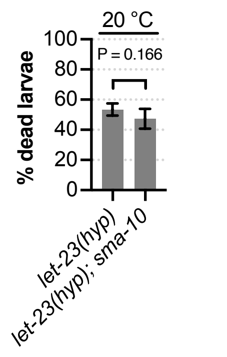

SUPPLEMENTARY FIGURE 4

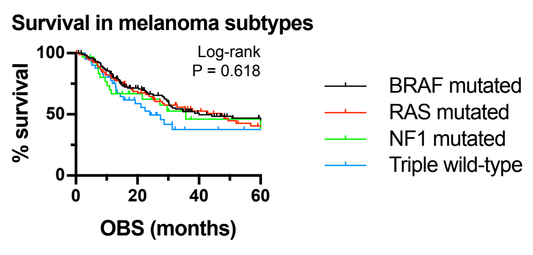

SUPPLEMENTARY FIGURE 5

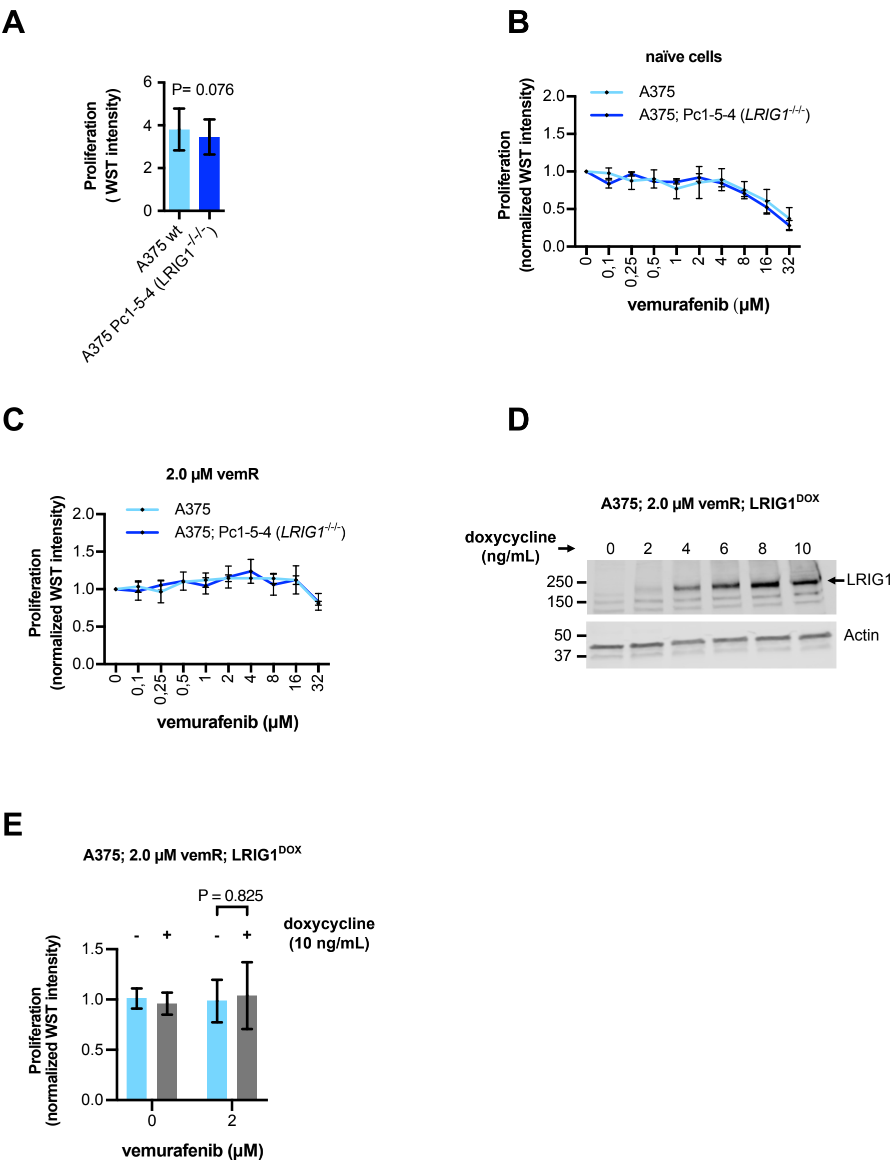

Supplement: Supplementary file 1 — Supplementary figures [file 41388_2021_1808_MOESM1_ESM.pdf]
