# Supplementary material for: LRIG1 is a conserved EGFR regulator involved in melanoma development, survival and treatment resistance
Source: Oncogene. 2021 May 4;40(21):3707–18. doi: 10.1038/s41388-021-01808-3 (PMC8154585; doi:10.1038/s41388-021-01808-3)
Supplement: Supplementary file 2 — Supplementary methods and figure legends [file 41388_2021_1808_MOESM2_ESM.pdf]

SUPPLEMENTARY METHODS

LRIG1 knockdown, knockout and overexpressing cells

To generate genetic knockdown and knockout of *LRIG1* in A375 melanoma cells, we ordered a plasmid containing two sgRNAs targeting both strands of *LRIG1* exon 11. sgRNAs were cloned into the pD1401-AD plasmid, which contains a Cas9(D10A)::GFP nickase sequence under the CMV promoter (Atum, Newark, CA, USA). The plasmid was transfected using Lipofectamine 2000 (Thermo Fisher Scientific, Waltham, MA, USA) according to the manufacturers protocol. GFP-positive cells were then single sorted into 96-well plates containing DMEM + 10 % FBS + 2 % PEST using a BD FACSAria™ III sorter. Single cells were expanded and eventually split into two replicate 12 well plates. One of each replicate was then expanded to 6-well plates, washed in cold PBS and scraped off in 200 µL 1x Tris-HCl, pH 8.0. 50 µL of each cell suspension was first lysed in a thermocycler and then treated with proteinase K. Lysates were then PCR screened for large indels and insertions (Fw primer sequence: cattccatgggcttgtgttg, Rv primer sequence: ccactaccattaatcagac). One clone that lacked the 278 bp wild-type band was then PCR amplified using primers flanking exon 11 (Fw primer sequence: gtttgactctaactctgttg, Rv primer sequence: gcataatgcaattgcagaag). Each of the three resulting bands were purified and cloned into a TOPO vector, sequenced and found to contain three copies of *LRIG1*: One had a deletion and the second had an insertion, both frameshifting. The third copy had a silent intronic insertion, where both pam sequences were intact. This clone was named Pc1-5. By repeating the entire process on Pc1-5 one more time we then isolated the Pc1-5-4 subclone, which was found to contain an additional insertion close to the splice acceptor site at the intron 10/exon 11 boundary.

LRIG1-inducible A375 and H1975 lines were generated by transduction with a doxycycline-inducible LRIG1::Flag construct as described previously[1]. A375 cells with constitutive overexpression of LRIG1 (LRIG1 oe) were generated by transducing a stably expressing full-length LRIG1 construct[2] under the CMV promoter. For shRNA, A375 and H1975 cells were

transduced with lentiviral particles generated from the SHC201 base plasmid (sh-control) or from an SHC201-based plasmid containing a short hairpin directed against LRIG1 (shLRIG1) (Sigma-Aldrich Sweden AB). All transduced cells were selected in 1.0 µg/ml puromycin and allowed to go through one passage without puromycin before use.

Growth factor stimulation experiments

Cells were starved in serum-free DMEM for 24 hours, followed by stimulation in DMEM + 10 ng/mL EGF for indicated durations of time (Figure 2F), or for 30 minutes in 1 µg/ml EGF or 10 % FBS +/- 5 µg/mL control peptide (His-MBP-Strep) or recombinant sLRIG1 as indicated (Figure 7). Experiments on vemurafenib-resistant cells were performed in the presence of 1 µM vemurafenib.

Antibodies

The monoclonal LRIG1 antibody 1A8 was generated by immunizing mice with recombinant LRIG1 ectodomain followed by fusion of the spleen cells with Sp2/0 cells. The resulting hybridomas were cloned and screened for reactivity against the immunizing antigen through ELISA and against cell surface expressed LRIG1 via flow cytometry. The positive clone 1A8 was shown to specifically recognize the leucine-rich repeats domain of LRIG1 through its ability to immunoprecipitate the corresponding recombinant fragment of human LRIG1. The 1A8 antibody was used at 4.8 mg/ml for immunohistochemistry (Supplementary figure 1A) and at 2.5 mg/ml for immunocytochemistry (Supplementary figure 1C, D). For immunocytochemistry, we used anti-FLAG M2 (Sigma-Aldrich, catalog no. F3165) at 8.0 mg/ml and anti-LRIG1 antibody Vina[49] (Agrisera AB, Vännäs, Sweden) at 5.0 mg/ml dilution.

Immunocytochemistry

To generate cell pellets, we first grew cells in T75 flasks until semi-confluent and with addition of 1 µg/ml doxycycline where indicated. Cells were then trypsinated, washed in PBS,

pelleted and fixed in 4 % phosphate-buffered paraformaldehyde (pH 7,3) for one hour under constant agitation. Cells were again pelleted and embedded in 3 % agarose/TBE before they were embedded in paraffine. Paraffine-embedded cell pellets were sectioned and stained using the same protocol as for tissue sections except that they were instead developed using a Vectastain Elite DAB Substrate kit (Vector Laboritories Inc, Burlingame, CA, USA). Slides were visualized on an Olympus BX51 microscope (Olympus, Tokyo, Japan).

LRIG1-inducible H1975 cells were seeded on glass coverslips in 6 well plates followed by cultivation, with or without 1 μg/ml of the inducer doxycycline, for three days. Thereafter, the cells were fixated with 4 % paraformaldehyde for 10 minutes or 24 hours. The fixated cells were immunostained using the Dako EnVision+ System-HRP (DAB) kit (Dako, Glostrup, Denmark; catalog no. K4006). Cell nuclei were counterstained with haematoxylin. The stained cells were visualized using a Panoramic 250 Flash scanner (3DHISTECH Ltd, Budapest, Hungary) or an Olympus BX51 microscope (Olympus, Tokyo, Japan).

CRISPR-Cas9-directed homologous recombination in C. elegans

The *lin-45(gf)* mutant allele kir1 was generated using CRISPR/Cas9-based genome editing as described previously[3] and was crossed out two times to the wild type N2 genome to decrease the before analysis. The sgRNA sequence was aaaaauuggagauuucggcuguuuuagagcuaugcu and the ssDNA repair template sequence was tgtcaacggtaaaaattggagatttcggccttgcaactgagaaaacgaaatggacagtgaacggaggtcaacagcaacaa. Recombinant Cas9 protein, tracrRNA, sgRNA and ssDNA were ordered (Integrated DNA Technologies, Inc., Coralville, IA, USA). Presence of the recombined DNA was followed by PCR (Fp: ctctccaaatactggccg, Rp: ctatcgtggagaattcttcg), followed by a DdeI restriction digest.

SUPPLEMENTARY FIGURE LEGENDS

Supplementary figure 1.

A) Tissue sections from normal skin, nevus, primary melanoma and metastatic melanoma stained with the 1A8 anti-LRIG1 antibody. Scale bars are 100 mm. **B)** Cartoon depicting epitopes of the LRIG1 antibodies used in this study. The reference protein is the full-length LRIG1::Flag protein translated from the doxycycline-inducible LRIG1 construct. **C)** Immunocytochemistry with three different LRIG1 antibodies on cell pellets from LRIG1-depleted cell lines with- or without forced expression of a full-length LRIG1::Flag construct. **D)** Immunocytochemistry with three different antibodies on H1975 cells grown on coverslips and fixed in PFA for the indicated time. Cells were grown with or without doxycycline-induced (DOX) expression of full-length LRIG1::Flag. In all cases, the nuclear immunoreactivity of LRIG1 disappeared with prolonged PFA fixation.

Supplementary figure 2.

Magnification of the phospho RTK signals that were significantly altered in A375 Pc1-5-4 [LRIG1$^{(-/-/-)}$]-cells compared to A375 parental cells. The dots are from a representative phospho-RTK array corresponding to Figure 2B-C.

Supplementary figure 3.

Percent dead larvae generated at 20 °C in hypomorph *let-23(n1045hyp)* and *let-23(n1045hyp); sma-10(wk89)* genotypes respectively. Error bars show +/- SD from three independent experiments. Statistical significance between genotypes was determined by a two-tailed Student's t-test.

Supplementary figure 4.

Kaplan-Meier survival plot showing observed survival interval (OBS) in metastasized patients, categorized into molecular subtypes as defined previously[4]. The subtypes are *BRAF* mutated (patients with activating V600 or K601 mutations in BRAF), *RAS* mutated (patients with activating G12, G13 or Q61 mutations in *HRAS, KRAS* or *NRAS*), *NF1*

mutated (patients with inactivating mutations in NF1) and triple wild-type (patients without any of the above mutations).

Supplementary figure 5.

A) The graph shows normalized quantification of WST intensity as a measure of proliferation in A375 wt and LRIG1 knockout cells +/- SD from 27 independent experiments. The P-value was calculated using a paired, two-tailed student's t-test. **BC)** Normalized quantifications of cell proliferation (WST intensity) after vemurafenib treatment in **B)** naïve A375 and A375; LRIG1 knockout cells and in **C)** vemurafenib-resistant lines. Error bars in **B)** and **C)** show +/- SD from three independent experiments respectively. **D)** Western blot showing LRIG1 expression in a vemurafenib-resistant line transduced with a dox-inducible cassette for LRIG1 expression. **E)** Normalized quantifications of cell proliferation (WST intensity) in a vemurafenib-resistant line with dox-inducible LRIG1 expression. Error bars show +/- SD from three independent experiments. Normalization in **B)**, **C)** and **E)** was done by dividing all measurements for an individual cell line with the mean WST intensity of two untreated conditions for that cell line.

SUPPLEMENTARY REFERENCES

1 Mao F, Holmlund C, Faraz M, Wang W, Bergenheim T, Kvarnbrink S *et al.* Lrig1 is a haploinsufficient tumor suppressor gene in malignant glioma. *Oncogenesis* 2018; **7**: 13.

2 Laederich MB, Funes-Duran M, Yen L, Ingalla E, Wu X, Carraway KL *et al.* The leucine-rich repeat protein LRIG1 is a negative regulator of ErbB family receptor tyrosine kinases. *J Biol Chem* 2004; **279**: 47050–47056.

3 Dokshin GA, Ghanta KS, Piscopo KM, Mello CC. Robust Genome Editing with Short Single-Stranded and Long, Partially Single-Stranded DNA Donors in Caenorhabditis elegans. *Genetics* 2018; **210**: 781–787.

4 Genomic Classification of Cutaneous Melanoma. *Cell* 2015; **161**: 1681–1696.
